# Using Deep Learning for Image-Based Plant Disease Detection

**DOI:** 10.3389/fpls.2016.01419

**Published:** 2016-09-22

**Authors:** Sharada P. Mohanty, David P. Hughes, Marcel Salathé

**Affiliations:** ^1^Digital Epidemiology Lab, EPFL Geneva, Switzerland; ^2^School of Life Sciences, EPFL Lausanne, Switzerland; ^3^School of Computer and Communication Sciences, EPFL Lausanne, Switzerland; ^4^Department of Entomology, College of Agricultural Sciences, Penn State University State College, PA, USA; ^5^Department of Biology, Eberly College of Sciences, Penn State University State College, PA, USA; ^6^Center for Infectious Disease Dynamics, Huck Institutes of Life Sciences, Penn State University State College, PA, USA

**Keywords:** crop diseases, machine learning, deep learning, digital epidemiology

## Abstract

Crop diseases are a major threat to food security, but their rapid identification remains difficult in many parts of the world due to the lack of the necessary infrastructure. The combination of increasing global smartphone penetration and recent advances in computer vision made possible by deep learning has paved the way for smartphone-assisted disease diagnosis. Using a public dataset of 54,306 images of diseased and healthy plant leaves collected under controlled conditions, we train a deep convolutional neural network to identify 14 crop species and 26 diseases (or absence thereof). The trained model achieves an accuracy of 99.35% on a held-out test set, demonstrating the feasibility of this approach. Overall, the approach of training deep learning models on increasingly large and publicly available image datasets presents a clear path toward smartphone-assisted crop disease diagnosis on a massive global scale.

## Introduction

Modern technologies have given human society the ability to produce enough food to meet the demand of more than 7 billion people. However, food security remains threatened by a number of factors including climate change (Tai et al., 2014), the decline in pollinators (Report of the Plenary of the Intergovernmental Science-PolicyPlatform on Biodiversity Ecosystem and Services on the work of its fourth session, 2016), plant diseases (Strange and Scott, 2005), and others. Plant diseases are not only a threat to food security at the global scale, but can also have disastrous consequences for smallholder farmers whose livelihoods depend on healthy crops. In the developing world, more than 80 percent of the agricultural production is generated by smallholder farmers (UNEP, 2013), and reports of yield loss of more than 50% due to pests and diseases are common (Harvey et al., 2014). Furthermore, the largest fraction of hungry people (50%) live in smallholder farming households (Sanchez and Swaminathan, 2005), making smallholder farmers a group that's particularly vulnerable to pathogen-derived disruptions in food supply.

Various efforts have been developed to prevent crop loss due to diseases. Historical approaches of widespread application of pesticides have in the past decade increasingly been supplemented by integrated pest management (IPM) approaches (Ehler, 2006). Independent of the approach, identifying a disease correctly when it first appears is a crucial step for efficient disease management. Historically, disease identification has been supported by agricultural extension organizations or other institutions, such as local plant clinics. In more recent times, such efforts have additionally been supported by providing information for disease diagnosis online, leveraging the increasing Internet penetration worldwide. Even more recently, tools based on mobile phones have proliferated, taking advantage of the historically unparalleled rapid uptake of mobile phone technology in all parts of the world (ITU, 2015).

**Figure 1:**
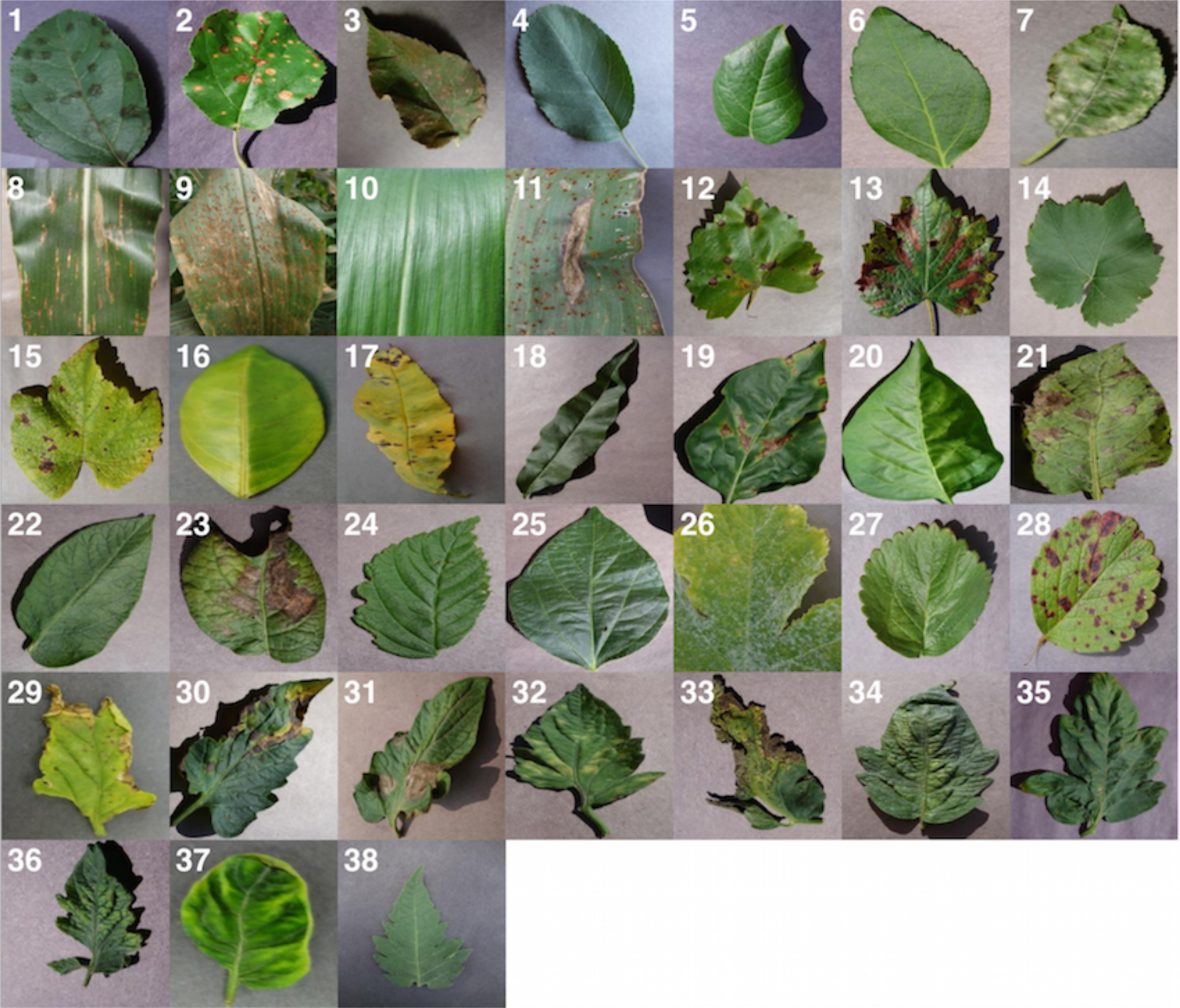
**Example of leaf images from the PlantVillage dataset, representing every crop-disease pair used. (1)** Apple Scab, *Venturia inaequalis* **(2)** Apple Black Rot, *Botryosphaeria obtusa* **(3)** Apple Cedar Rust, *Gymnosporangium juniperi-virginianae* **(4)** Apple healthy **(5)** Blueberry healthy **(6)** Cherry healthy **(7)** Cherry Powdery Mildew, *Podoshaera clandestine* **(8)** Corn Gray Leaf Spot, *Cercospora zeae-maydis* **(9)** Corn Common Rust, *Puccinia sorghi* **(10)** Corn healthy **(11)** Corn Northern Leaf Blight, *Exserohilum turcicum* **(12)** Grape Black Rot, *Guignardia bidwellii*, **(13)** Grape Black Measles (Esca), *Phaeomoniella aleophilum, Phaeomoniella chlamydospora* **(14)** Grape Healthy **(15)** Grape Leaf Blight, *Pseudocercospora vitis* **(16)** Orange Huanglongbing (Citrus Greening), *Candidatus Liberibacter spp.* **(17)** Peach Bacterial Spot, *Xanthomonas campestris* **(18)** Peach healthy **(19)** Bell Pepper Bacterial Spot, *Xanthomonas campestris* **(20)** Bell Pepper healthy **(21)** Potato Early Blight, *Alternaria solani* **(22)** Potato healthy **(23)** Potato Late Blight, *Phytophthora infestans* **(24)** Raspberry healthy **(25)** Soybean healthy **(26)** Squash Powdery Mildew, *Erysiphe cichoracearum* **(27)** Strawberry Healthy **(28)** Strawberry Leaf Scorch, *Diplocarpon earlianum* **(29)** Tomato Bacterial Spot, *Xanthomonas campestris pv. vesicatoria* **(30)** Tomato Early Blight, *Alternaria solani* **(31)** Tomato Late Blight, *Phytophthora infestans* **(32)** Tomato Leaf Mold, *Passalora fulva* **(33)** Tomato Septoria Leaf Spot, *Septoria lycopersici* **(34)** Tomato Two Spotted Spider Mite, *Tetranychus urticae* **(35)** Tomato Target Spot, *Corynespora cassiicola* **(36)** Tomato Mosaic Virus **(37)** Tomato Yellow Leaf Curl Virus **(38)** Tomato healthy.

Smartphones in particular offer very novel approaches to help identify diseases because of their computing power, high-resolution displays, and extensive built-in sets of accessories, such as advanced HD cameras. It is widely estimated that there will be between 5 and 6 billion smartphones on the globe by 2020. At the end of 2015, already 69% of the world's population had access to mobile broadband coverage, and mobile broadband penetration reached 47% in 2015, a 12-fold increase since 2007 (ITU, 2015). The combined factors of widespread smartphone penetration, HD cameras, and high performance processors in mobile devices lead to a situation where disease diagnosis based on automated image recognition, if technically feasible, can be made available at an unprecedented scale. Here, we demonstrate the technical feasibility using a deep learning approach utilizing 54,306 images of 14 crop species with 26 diseases (or healthy) made openly available through the project PlantVillage (Hughes and Salathé, 2015). An example of each crop—disease pair can be seen in Figure 1.

Computer vision, and object recognition in particular, has made tremendous advances in the past few years. The PASCAL VOC Challenge (Everingham et al., 2010), and more recently the Large Scale Visual Recognition Challenge (ILSVRC) (Russakovsky et al., 2015) based on the ImageNet dataset (Deng et al., 2009) have been widely used as benchmarks for numerous visualization-related problems in computer vision, including object classification. In 2012, a large, deep convolutional neural network achieved a top-5 error of 16.4% for the classification of images into 1000 possible categories (Krizhevsky et al., 2012). In the following 3 years, various advances in deep convolutional neural networks lowered the error rate to 3.57% (Krizhevsky et al., 2012; Simonyan and Zisserman, 2014; Zeiler and Fergus, 2014; He et al., 2015; Szegedy et al., 2015). While training large neural networks can be very time-consuming, the trained models can classify images very quickly, which makes them also suitable for consumer applications on smartphones.

Deep neural networks have recently been successfully applied in many diverse domains as examples of end to end learning. Neural networks provide a mapping between an input—such as an image of a diseased plant—to an output—such as a crop~disease pair. The nodes in a neural network are mathematical functions that take numerical inputs from the incoming edges, and provide a numerical output as an outgoing edge. Deep neural networks are simply mapping the input layer to the output layer over a series of stacked layers of nodes. The challenge is to create a deep network in such a way that both the structure of the network as well as the functions (nodes) and edge weights correctly map the input to the output. Deep neural networks are trained by tuning the network parameters in such a way that the mapping improves during the training process. This process is computationally challenging and has in recent times been improved dramatically by a number of both conceptual and engineering breakthroughs (LeCun et al., 2015; Schmidhuber, 2015).

In order to develop accurate image classifiers for the purposes of plant disease diagnosis, we needed a large, verified dataset of images of diseased and healthy plants. Until very recently, such a dataset did not exist, and even smaller datasets were not freely available. To address this problem, the PlantVillage project has begun collecting tens of thousands of images of healthy and diseased crop plants (Hughes and Salathé, 2015), and has made them openly and freely available. Here, we report on the classification of 26 diseases in 14 crop species using 54,306 images with a convolutional neural network approach. We measure the performance of our models based on their ability to predict the correct crop-diseases pair, given 38 possible classes. The best performing model achieves a mean F_1_ score of 0.9934 (overall accuracy of 99.35%), hence demonstrating the technical feasibility of our approach. Our results are a first step toward a smartphone-assisted plant disease diagnosis system.

## Methods

### Dataset description

We analyze 54,306 images of plant leaves, which have a spread of 38 class labels assigned to them. Each class label is a crop-disease pair, and we make an attempt to predict the crop-disease pair given just the image of the plant leaf. Figure 1 shows one example each from every crop-disease pair from the PlantVillage dataset. In all the approaches described in this paper, we resize the images to 256 × 256 pixels, and we perform both the model optimization and predictions on these downscaled images.

Across all our experiments, we use three different versions of the whole PlantVillage dataset. We start with the PlantVillage dataset as it is, in color; then we experiment with a gray-scaled version of the PlantVillage dataset, and finally we run all the experiments on a version of the PlantVillage dataset where the leaves were segmented, hence removing all the extra background information which might have the potential to introduce some inherent bias in the dataset due to the regularized process of data collection in case of PlantVillage dataset. Segmentation was automated by the means of a script tuned to perform well on our particular dataset. We chose a technique based on a set of masks generated by analysis of the color, lightness and saturation components of different parts of the images in several color spaces (Lab and HSB). One of the steps of that processing also allowed us to easily fix color casts, which happened to be very strong in some of the subsets of the dataset, thus removing another potential bias.

**Figure 2:**
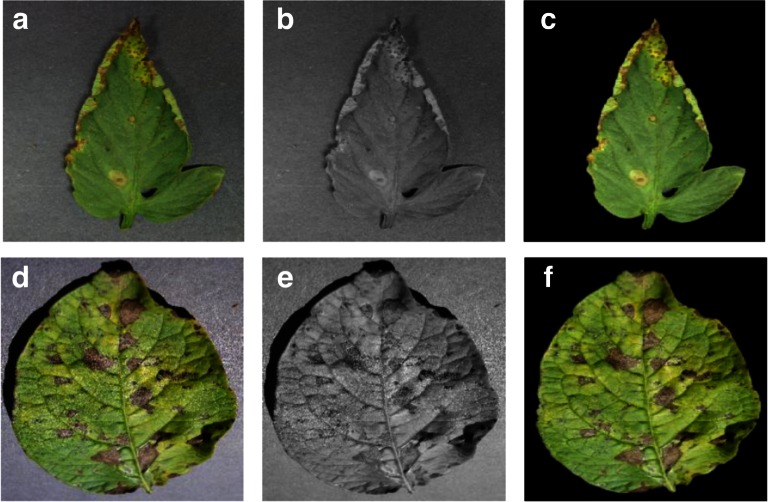
**Sample images from the three different versions of the PlantVillage dataset used in various experimental configurations. (A)** Leaf 1 color, **(B)** Leaf 1 grayscale, **(C)** Leaf 1 segmented, **(D)** Leaf 2 color, **(E)** Leaf 2 gray-scale, **(F)** Leaf 2 segmented.

This set of experiments was designed to understand if the neural network actually learns the “notion” of plant diseases, or if it is just learning the inherent biases in the dataset. Figure 2 shows the different versions of the same leaf for a randomly selected set of leaves.

### Measurement of performance

To get a sense of how our approaches will perform on new unseen data, and also to keep a track of if any of our approaches are overfitting, we run all our experiments across a whole range of train-test set splits, namely 80–20 (80% of the whole dataset used for training, and 20% for testing), 60–40 (60% of the whole dataset used for training, and 40% for testing), 50–50 (50% of the whole dataset used for training, and 50% for testing), 40–60 (40% of the whole dataset used for training, and 60% for testing) and finally 20–80 (20% of the whole dataset used for training, and 80% for testing). It must be noted that in many cases, the PlantVillage dataset has multiple images of the same leaf (taken from different orientations), and we have the mappings of such cases for 41,112 images out of the 54,306 images; and during all these test-train splits, we make sure all the images of the same leaf goes either in the training set or the testing set. Further, for every experiment, we compute the mean precision, mean recall, mean F_1_ score, along with the overall accuracy over the whole period of training at regular intervals (at the end of every epoch). We use the final mean F_1_ score for the comparison of results across all of the different experimental configurations.

### Approach

We evaluate the applicability of deep convolutional neural networks for the classification problem described above. We focus on two popular architectures, namely AlexNet (Krizhevsky et al., 2012), and GoogLeNet (Szegedy et al., 2015), which were designed in the context of the “Large Scale Visual Recognition Challenge” (ILSVRC) (Russakovsky et al., 2015) for the ImageNet dataset (Deng et al., 2009).

The AlexNet architecture (see Figure S2) follows the same design pattern as the LeNet-5 (LeCun et al., 1989) architecture from the 1990s. The LeNet-5 architecture variants are usually a set of stacked convolution layers followed by one or more fully connected layers. The convolution layers optionally may have a normalization layer and a pooling layer right after them, and all the layers in the network usually have ReLu non-linear activation units associated with them. AlexNet consists of 5 convolution layers, followed by 3 fully connected layers, and finally ending with a softMax layer. The first two convolution layers (conv{1, 2}) are each followed by a normalization and a pooling layer, and the last convolution layer (conv5) is followed by a single pooling layer. The final fully connected layer (fc8) has 38 outputs in our adapted version of AlexNet (equaling the total number of classes in our dataset), which feeds the softMax layer. The softMax layer finally exponentially normalizes the input that it gets from (fc8), thereby producing a distribution of values across the 38 classes that add up to 1. These values can be interpreted as the confidences of the network that a given input image is represented by the corresponding classes. All of the first 7 layers of AlexNet have a ReLu non-linearity activation unit associated with them, and the first two fully connected layers (fc{6, 7}) have a dropout layer associated with them, with a dropout ratio of 0.5.

The GoogleNet architecture on the other hand is a much deeper and wider architecture with 22 layers, while still having considerably lower number of parameters (5 million parameters) in the network than AlexNet (60 million parameters). An application of the “network in network” architecture (Lin et al., 2013) in the form of the inception modules is a key feature of the GoogleNet architecture. The inception module uses parallel 1 × 1, 3 × 3, and 5 × 5 convolutions along with a max-pooling layer in parallel, hence enabling it to capture a variety of features in parallel. In terms of practicality of the implementation, the amount of associated computation needs to be kept in check, which is why 1 × 1 convolutions before the above mentioned 3 × 3, 5 × 5 convolutions (and also after the max-pooling layer) are added for dimensionality reduction. Finally, a filter concatenation layer simply concatenates the outputs of all these parallel layers. While this forms a single inception module, a total of 9 inception modules is used in the version of the GoogLeNet architecture that we use in our experiments. A more detailed overview of this architecture can be found for reference in (Szegedy et al., 2015).

We analyze the performance of both these architectures on the PlantVillage dataset by training the model from scratch in one case, and then by adapting already trained models (trained on the ImageNet dataset) using transfer learning. In case of transfer learning, we re-initialize the weights of layer fc8 in case of AlexNet, and of the loss {1,2,3}/classifier layers in case of GoogLeNet. Then, when training the model, we do not limit the learning of any of the layers, as is sometimes done for transfer learning. In other words, the key difference between these two learning approaches (transfer vs. training from scratch) is in the initial state of weights of a few layers, which lets the transfer learning approach exploit the large amount of visual knowledge already learned by the pre-trained AlexNet and GoogleNet models extracted from ImageNet (Russakovsky et al., 2015).

To summarize, we have a total of 60 experimental configurations, which vary on the following parameters:

1. **Choice of deep learning architecture:**
  AlexNet,
  GoogLeNet.
2. **Choice of training mechanism:**
  Transfer Learning,
  Training from Scratch.
3. **Choice of dataset type:**
  Color,
  Gray scale,
  Leaf Segmented.
4. **Choice of training-testing set distribution:**
  Train: 80%, Test: 20%,
  Train: 60%, Test: 40%,
  Train: 50%, Test: 50%,
  Train: 40%, Test: 60%,
  Train: 20%, Test: 80%.

Throughout this paper, we have used the notation of *Architecture:TrainingMechanism:DatasetType:Train-Test-Set-Distribution* to refer to particular experiments. For instance, to refer to the experiment using the GoogLeNet architecture, which was trained using transfer learning on the gray-scaled PlantVillage dataset on a train—test set distribution of 60–40, we will use the notation *GoogLeNet:TransferLearning:GrayScale:60–40*.

**Figure 3:**
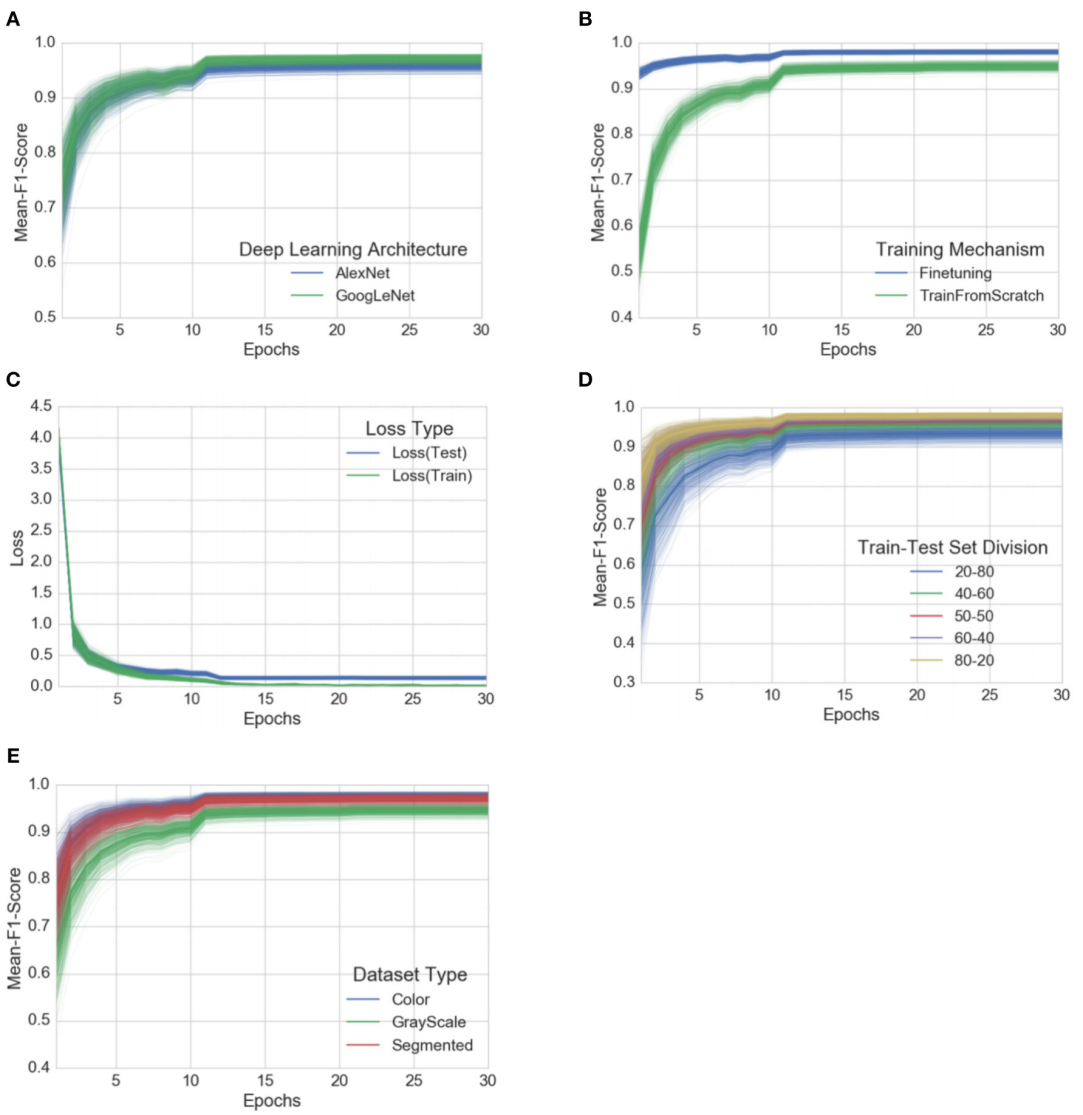
**Progression of mean F_1_ score and loss through the training period of 30 epochs across all experiments, grouped by experimental configuration parameters**. The intensity of a particular class at any point is proportional to the corresponding uncertainty across all experiments with the particular configurations. **(A)** Comparison of progression of mean F_1_ score across all experiments, grouped by deep learning architecture, **(B)** Comparison of progression of mean F_1_ score across all experiments, grouped by training mechanism, **(C)** Comparison of progression of train-loss and test-loss across all experiments, **(D)** Comparison of progression of mean F_1_ score across all experiments, grouped by train-test set splits, **(E)** Comparison of progression of mean F_1_ score across all experiments, grouped by dataset type. A similar plot of all the observations, as it is, across all the experimental configurations can be found in the Supplementary Material.

Each of these 60 experiments runs for a total of 30 epochs, where one epoch is defined as the number of training iterations in which the particular neural network has completed a full pass of the whole training set. The choice of 30 epochs was made based on the empirical observation that in all of these experiments, the learning always converged well within 30 epochs (as is evident from the aggregated plots (Figure 3) across all the experiments).

To enable a fair comparison between the results of all the experimental configurations, we also tried to standardize the hyper-parameters across all the experiments, and we used the following hyper-parameters in all of the experiments:

- Solver type: Stochastic Gradient Descent,
- Base learning rate: 0.005,
- Learning rate policy: Step (decreases by a factor of 10 every 30/3 epochs),
- Momentum: 0.9,
- Weight decay: 0.0005,
- Gamma: 0.1,
- Batch size: 24 (in case of GoogLeNet), 100 (in case of AlexNet).

All the above experiments were conducted using our own fork of Caffe (Jia et al., 2014), which is a fast, open source framework for deep learning. The basic results, such as the overall accuracy can also be replicated using a standard instance of caffe.

## Results

At the outset, we note that on a dataset with 38 class labels, random guessing will only achieve an overall accuracy of 2.63% on average. Across all our experimental configurations, which include three visual representations of the image data (see Figure 2), the overall accuracy we obtained on the PlantVillage dataset varied from 85.53% (in case of *AlexNet::TrainingFromScratch::GrayScale::80–20*) to 99.34% (in case of *GoogLeNet::TransferLearning::Color::80–20*), hence showing strong promise of the deep learning approach for similar prediction problems. Table 1 shows the mean F_1_ score, mean precision, mean recall, and overall accuracy across all our experimental configurations. All the experimental configurations run for a total of 30 epochs each, and they almost consistently converge after the first step down in the learning rate.

**Table 1:** **Mean F_1_ score across various experimental configurations at the end of 30 epochs**.

	**AlexNet**		**GoogleNet**	
	**Transfer learning**	**Training from scratch**	**Transfer learning**	**Training from scratch**
**TRAIN: 200%, TEST: 80%**				
Color	0.9736_{0.9742, 0.9737, 0.9738}_	0.9118_{0.9137, 0.9132, 0.9130}_	**0.9820**_{0.9824, 0.9821, 0.9821}_	0.9430_{0.9440, 0.9431, 0.9429}_
Grayscale	0.9361_{0.9368, 0.9369, 0.9371}_	0.8524_{0.8539, 0.8555, 0.8553}_	**0.9563**_{0.9570, 0.9564, 0.9564}_	0.8828_{0.8842, 0.6835,0.8841}_
Segmented	0.9724_{0.9727, 0.9727, 0.9726}_	0.8945_{0.8956, 0.8963, 0.8969}_	**0.9808**_{0.9810, 0.9808, 0.9808}_	0.9377_{0.9388, 0.9380, 0.9380}_
**TRAIN: 400%, TEST: 60%**				
Color	0.9860_{0.9861, 0.9861, 0.9860}_	0.9555_{0.9557, 0.9558, 0.9558}_	**0.9914**_{0.9914, 0.9914, 0.9914}_	0.9729_{0.9731, 0.9729, 0.9729}_
Grayscale	0.9584_{0.9588, 0.9589, 0.9588}_	0.9088_{0.9090, 0.9101, 0.9100}_	**0.9714**_{0.9717, 0.9716, 0.9716}_	0.9361_{0.9364, 0.9363, 0.9364}_
Segmented	0.9812_{0.9814, 0.9813, 0.9813}_	0.9404_{0.9409, 0.9408, 0.9408}_	**0.9896**_{0.9896, 0.9896, 0.9898}_	0.9643_{0.9647, 0.9642, 0.9642}_
**TRAIN: 50%, TEST: 50%**				
Color	0.9896_{0.9897, 0.9896, 0.9897}_	0.9644_{0.9647, 0.9647, 0.9647}_	**0.9916**_{0.9916, 0.9916, 0.9916}_	0.9772_{0.9774, 0.9773, 0.9773}_
Grayscale	0.9661_{0.9663, 0.9663, 0.9663}_	0.9312_{0.9315, 0.9318, 0.9319}_	**0.9788**_{0.9789, 0.9788, 0.9788}_	0.9507_{0.9510, 0.9507, 0.9509}_
Segmented	0.9867_{0.9868, 0.9868, 0.9869}_	0.9551_{0.9552, 0.9555, 0.9556}_	**0.9909**_{0.9910, 0.9910, 0.9910}_	0.9720_{0.9721, 0.9721, 0.9722}_
**TRAIN: 600%, TEST: 40%**				
Color	0.9907_{0.9908, 0.9908, 0.9907}_	0.9724_{0.9725, 0.9725, 0.9725}_	**0.9924**_{0.9924, 0.9924, 0.9924}_	0.9824_{0.9825, 0.9824, 0.9824}_
Grayscale	0.9686_{0.9689, 0.9688, 0.9688}_	0.9388_{0.9396, 0.9395, 0.9391}_	**0.9785**_{0.9789, 0.9786, 0.9787}_	0.9547_{0.9554, 0.9548, 0.9551}_
Segmented	0.9855_{0.9856, 0.9856, 0.9856}_	0.9595_{0.9597, 0.9597, 0.9596}_	**0.9905{**_0.9906, 0.9906, 0.9906}_	0.9740_{0.9743, 0.9740, 0.9745}_
**TRAIN: 80%, TEST: 20%**				
Color	**0.9927**_{0.9928, 0.9927, 0.9928)_	**0.9782**_{0.9786, 0.9782, 0.9782}_	**0.9934**_{0.9935, 0.9935, 0.9935}_	**0.9836**_{0.9839, 0.9837, 0.9837}_
Grayscale	**0.9726**_{0.9728, 0.9727, 0.9725}_	**0.9449**_{0.9451, 0.9454, 0.9452}_	**0.9800**_{0.9804, 0.9801, 0.9798}_	**0.9621**_{0.9624, 0.9621, 0.9621}_
Segmented	**0.9891**_{0.9893, 0.9891, 0.9892}_	**0.9722**_{0.9725, 0.9724, 0.9723}_	**0.9925**_{0.9925, 0.9925, 0.9924}_	**0.9824**_{0.9827, 0.9824, 0.9822}_

Each cell in the table represents the mean F_1_ score_{mean precision, mean recall, overall accuracy}_ for the corresponding experimental configuration.

The bold values are the F_1_ scores of the best performing models in the respective row/column.

To address the issue of over-fitting, we vary the test set to train set ratio and observe that even in the extreme case of training on only 20% of the data and testing the trained model on the rest 80% of the data, the model achieves an overall accuracy of 98.21% (mean F_1_ score of 0.9820) in the case of *GoogLeNet::TransferLearning::Color::20–80*. As expected, the overall performance of both AlexNet and GoogLeNet do degrade if we keep increasing the test set to train set ratio (see Figure 3D), but the decrease in performance is not as drastic as we would expect if the model was indeed over-fitting. Figure 3C also shows that there is no divergence between the validation loss and the training loss, confirming that over-fitting is not a contributor to the results we obtain across all our experiments.

Among the AlexNet and GoogLeNet architectures, GoogLeNet consistently performs better than AlexNet (Figure 3A), and based on the method of training, transfer learning always yields better results (Figure 3B), both of which were expected.

The three versions of the dataset (color, gray-scale, and segmented) show a characteristic variation in performance across all the experiments when we keep the rest of the experimental configuration constant. The models perform the best in case of the colored version of the dataset. When designing the experiments, we were concerned that the neural networks might only learn to pick up the inherent biases associated with the lighting conditions, the method and apparatus of collection of the data. We therefore experimented with the gray-scaled version of the same dataset to test the model's adaptability in the absence of color information, and its ability to learn higher level structural patterns typical to particular crops and diseases. As expected, the performance did decrease when compared to the experiments on the colored version of the dataset, but even in the case of the worst performance, the observed mean F_1_ score was 0.8524 (overall accuracy of 85.53%). The segmented versions of the whole dataset was also prepared to investigate the role of the background of the images in overall performance, and as shown in Figure 3E, the performance of the model using segmented images is consistently better than that of the model using gray-scaled images, but slightly lower than that of the model using the colored version of the images.

**Figure 4:**
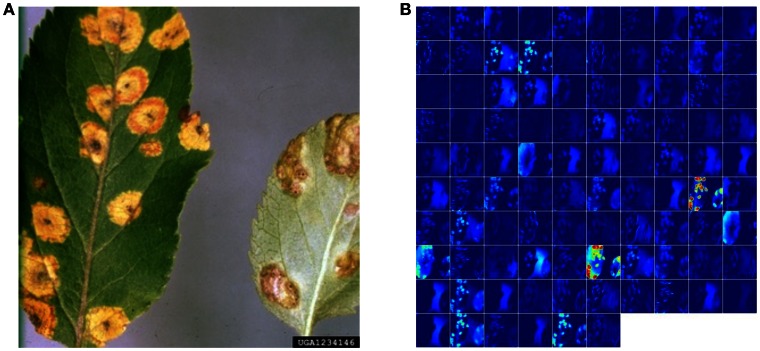
**Visualization of activations in the initial layers of an AlexNet architecture demonstrating that the model has learnt to efficiently activate against the diseased spots on the example leaf. (A)** Example image of a leaf suffering from Apple Cedar Rust, selected from the top-20 images returned by Bing Image search for the keywords “Apple Cedar Rust Leaves” on April 4th, 2016. Image Reference: Clemson University - USDA Cooperative Extension Slide Series, Bugwood. org. **(B)** Visualization of activations in the first convolution layer(conv1) of an AlexNet architecture trained using *AlexNet:Color:TrainFromScratch:80–20* when doing a forward pass on the image in shown in panel b.

While these approaches yield excellent results on the PlantVillage dataset which was collected in a controlled environment, we also assessed the model's performance on images sampled from trusted online sources, such as academic agriculture extension services. Such images are not available in large numbers, and using a combination of automated download from Bing Image Search and IPM Images with a visual verification step, we obtained two small, verified datasets of 121 (dataset 1) and 119 images (dataset 2), respectively (see Supplementary Material for a detailed description of the process). Using the best model on these datasets, we obtained an overall accuracy of 31.40% in dataset 1, and 31.69% in dataset 2, in successfully predicting the correct class label (i.e., crop and disease information) from among 38 possible class labels. We note that a random classifier will obtain an average accuracy of only 2.63%. Across all images, the correct class was in the top-5 predictions in 52.89% of the cases in dataset 1, and in 65.61% of the cases in dataset 2. The best models for the two datasets were *GoogLeNet:Segmented:TransferLearning:80–20* for dataset 1, and *GoogLeNet:Color:TransferLearning:80–20* for dataset 2. An example image from theses datasets, along with its visualization of activations in the initial layers of an AlexNet architecture, can be seen in Figure 4.

So far, all results have been reported under the assumption that the model needs to detect both the crop species and the disease status. We can limit the challenge to a more realistic scenario where the crop species is provided, as it can be expected to be known by those growing the crops. To assess this the performance of the model under this scenario, we limit ourselves to crops where we have at least *n* > = 2 (to avoid trivial classification) or *n* > = 3 classes per crop. In the *n* > = 2 case, dataset 1 contains 33 classes distributed among 9 crops. Random guessing in such a dataset would achieve an accuracy of 0.225, while our model has an accuracy of 0.478. In the *n* > = 3 case, the dataset contains 25 classes distributed among 5 crops. Random guessing in such a dataset would achieve an accuracy of 0.179, while our model has an accuracy of 0.411.

Similarly, in the *n* > = 2 case, dataset 2 contains 13 classes distributed among 4 crops. Random guessing in such a dataset would achieve an accuracy of 0.314, while our model has an accuracy of 0.545. In the *n* > = 3 case, the dataset contains 11 classes distributed among 3 crops. Random guessing in such a dataset would achieve an accuracy of 0.288, while our model has an accuracy of 0.485.

## Discussion

The performance of convolutional neural networks in object recognition and image classification has made tremendous progress in the past few years. (Krizhevsky et al., 2012; Simonyan and Zisserman, 2014; Zeiler and Fergus, 2014; He et al., 2015; Szegedy et al., 2015). Previously, the traditional approach for image classification tasks has been based on hand-engineered features, such as SIFT (Lowe, 2004), HoG (Dalal and Triggs, 2005), SURF (Bay et al., 2008), etc., and then to use some form of learning algorithm in these feature spaces. The performance of these approaches thus depended heavily on the underlying predefined features. Feature engineering itself is a complex and tedious process which needs to be revisited every time the problem at hand or the associated dataset changes considerably. This problem occurs in all traditional attempts to detect plant diseases using computer vision as they lean heavily on hand-engineered features, image enhancement techniques, and a host of other complex and labor-intensive methodologies.

In addition, traditional approaches to disease classification via machine learning typically focus on a small number of classes usually within a single crop. Examples include a feature extraction and classification pipeline using thermal and stereo images in order to classify tomato powdery mildew against healthy tomato leaves (Raza et al., 2015); the detection of powdery mildew in uncontrolled environments using RGB images (Hernández-Rabadán et al., 2014); the use of RGBD images for detection of apple scab (Chéné et al., 2012) the use of fluorescence imaging spectroscopy for detection of citrus huanglongbing (Wetterich et al., 2012) the detection of citrus huanglongbing using near infrared spectral patterns (Sankaran et al., 2011) and aircraft-based sensors (Garcia-Ruiz et al., 2013) the detection of tomato yellow leaf curl virus by using a set of classic feature extraction steps, followed by classification using a support vector machines pipeline (Mokhtar et al., 2015), and many others. A very recent review on the use of machine learning on plant phenotyping (Singh et al., 2015) extensively discusses the work in this domain. While neural networks have been used before in plant disease identification (Huang, 2007) (for the classification and detection of *Phalaenopsis* seedling disease like bacterial soft rot, bacterial brown spot, and *Phytophthora* black rot), the approach required representing the images using a carefully selected list of texture features before the neural network could classify them.

Our approach is based on recent work Krizhevsky et al. (2012) which showed for the first time that end-to-end supervised training using a deep convolutional neural network architecture is a practical possibility even for image classification problems with a very large number of classes, beating the traditional approaches using hand-engineered features by a substantial margin in standard benchmarks. The absence of the labor-intensive phase of feature engineering and the generalizability of the solution makes them a very promising candidate for a practical and scaleable approach for computational inference of plant diseases.

Using the deep convolutional neural network architecture, we trained a model on images of plant leaves with the goal of classifying both crop species and the presence and identity of disease on images that the model had not seen before. Within the PlantVillage data set of 54,306 images containing 38 classes of 14 crop species and 26 diseases (or absence thereof), this goal has been achieved as demonstrated by the top accuracy of 99.35%. Thus, without any feature engineering, the model correctly classifies crop and disease from 38 possible classes in 993 out of 1000 images. Importantly, while the training of the model takes a lot of time (multiple hours on a high performance GPU cluster computer), the classification itself is very fast (less than a second on a CPU), and can thus easily be implemented on a smartphone. This presents a clear path toward smartphone-assisted crop disease diagnosis on a massive global scale.

However, there are a number of limitations at the current stage that need to be addressed in future work. First, when tested on a set of images taken under conditions different from the images used for training, the model's accuracy is reduced substantially, to just above 31%. It's important to note that this accuracy is much higher than the one based on random selection of 38 classes (2.6%), but nevertheless, a more diverse set of training data is needed to improve the accuracy. Our current results indicate that more (and more variable) data alone will be sufficient to substantially increase the accuracy, and corresponding data collection efforts are underway.

The second limitation is that we are currently constrained to the classification of single leaves, facing up, on a homogeneous background. While these are straightforward conditions, a real world application should be able to classify images of a disease as it presents itself directly on the plant. Indeed, many diseases don't present themselves on the upper side of leaves only (or at all), but on many different parts of the plant. Thus, new image collection efforts should try to obtain images from many different perspectives, and ideally from settings that are as realistic as possible.

At the same time, by using 38 classes that contain both crop species and disease status, we have made the challenge harder than ultimately necessary from a practical perspective, as growers are expected to know which crops they are growing. Given the very high accuracy on the PlantVillage dataset, limiting the classification challenge to the disease status won't have a measurable effect. However, on the real world datasets, we can measure noticeable improvements in accuracy. Overall, the presented approach works reasonably well with many different crop species and diseases, and is expected to improve considerably with more training data.

Finally, it's worth noting that the approach presented here is not intended to replace existing solutions for disease diagnosis, but rather to supplement them. Laboratory tests are ultimately always more reliable than diagnoses based on visual symptoms alone, and oftentimes early-stage diagnosis via visual inspection alone is challenging. Nevertheless, given the expectation of more than 5 Billion smartphones in the world by 2020—of which almost a Billion in Africa (GSMA Intelligence, 2016)—we do believe that the approach represents a viable additional method to help prevent yield loss. What's more, in the future, image data from a smartphone may be supplemented with location and time information for additional improvements in accuracy. Last but not least, it would be prudent to keep in mind the stunning pace at which mobile technology has developed in the past few years, and will continue to do so. With ever improving number and quality of sensors on mobiles devices, we consider it likely that highly accurate diagnoses via the smartphone are only a question of time.

## Author contributions

MS, DH, and SM conceived the study and wrote the paper. SM implemented the algorithm described.

### Conflict of interest statement

The authors declare that the research was conducted in the absence of any commercial or financial relationships that could be construed as a potential conflict of interest.

## Resources

The data and the code used in this paper are available at the following locations:

Data: https://github.com/salathegroup/plantvillage_deep learning_paper_dataset

Code: https://github.com/salathegroup/plantvillage_deep learning_paper_analysis

More image data can be found at https://www.plantvillage.org/en/plant_images
