# Supplementary material for: Using Deep Learning for Image-Based Plant Disease Detection
Source: Front Plant Sci. 2016 Sep 22;7:1419. doi: 10.3389/fpls.2016.01419 (PMC5032846; doi:10.3389/fpls.2016.01419)
Supplement: Supplementary file 1 [file DataSheet1.docx]

**SUPPLEMENTARY MATERIAL**

In order to assess the performance of the model on a "real world" data set, we downloaded images from Bing Image search on April 4, 2016. For each of the 38 classes depicted in Figure 1 in the main text, we performed a search with the query "{crop and disease name} leaf leaves", where {crop and disease name} was replaced by the crop and disease name pairs given for each of the 38 classes in the caption of Figure 1 in the main text. Following this, we downloaded the top 10 images for each class. Then, for each image of plant diseases, we verified that the image was by a reputable source (mainly extension services or plant pathology websites of universities), and that it was showing leaves in approximately the same configuration i.e. showing at least one upper side of the most of one leaf. If this process left us with no images in that class, we did a manual search identifying at least one such image from a reputable source.

**Using SVM for disease classification on the PlantVillage dataset**

In order to get a comparative base line for the deep learning approach presented in the main text, we trained a SVM-based *Bag of Visual Words* model with SIFT features. Since the configuration of using the color data set split into 80% training and 20% testing yielded the best results, we used this data division for the SVM model as well. However, due to the computational complexity of SVM for a multi-class (38) classification problem, we trained it on only 20% of the training set (8,609 images obtained through random sampling), and then tested it against the entire testing set of 10,547 images. Doing this, we obtained an accuracy of 69.38%.

The code for the experiments using the SVM model is available at: https://github.com/salathegroup/Minimal-Bag-of-Visual-Words-Image-Classifier

**Figure S1
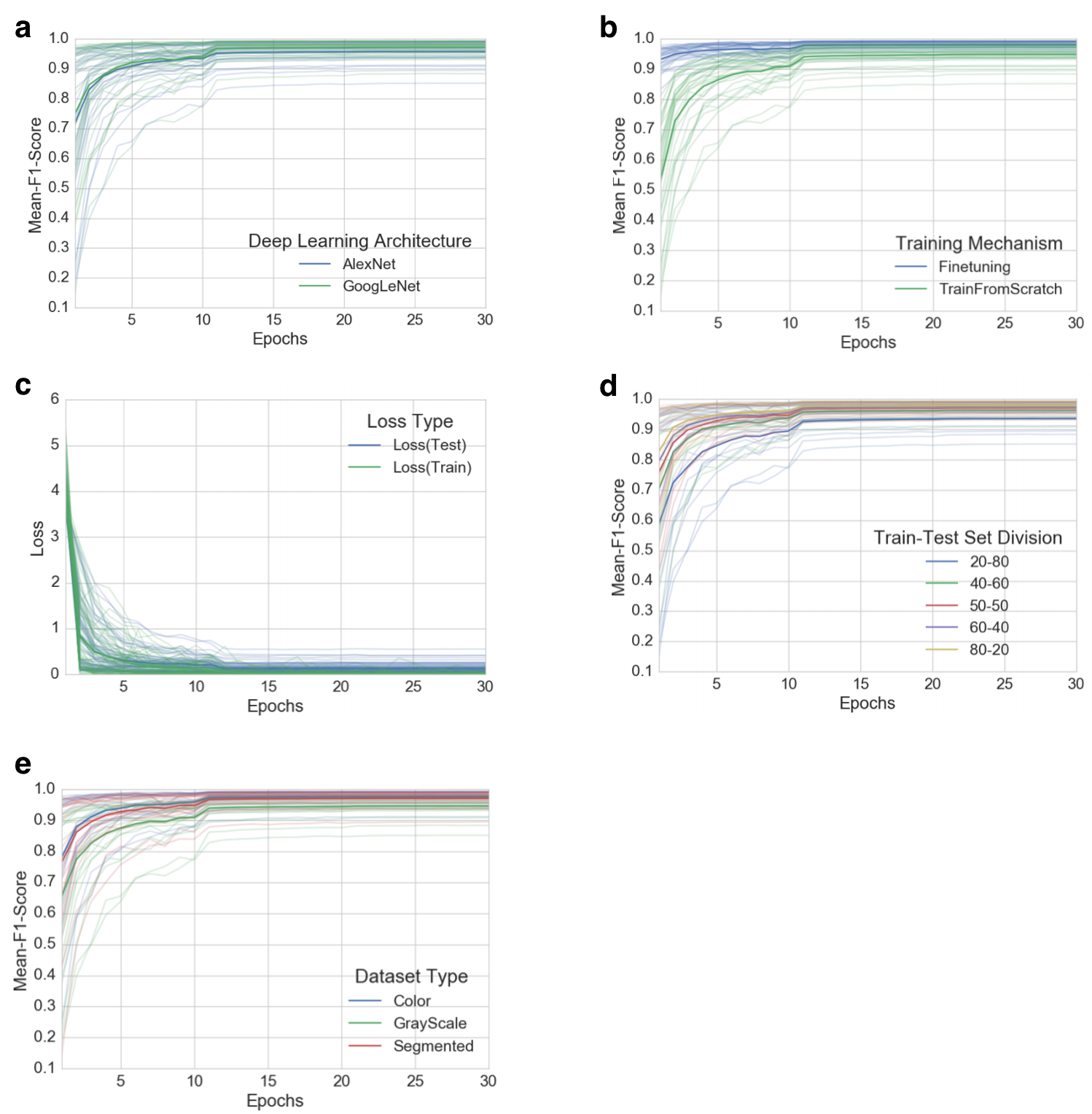
**

**Fig. S1.** Progression of mean F1 score and loss through the training period of 30 epochs across all experiments, grouped by experimental configuration parameters. a) Comparison of progression of mean F1 score across all experiments, grouped by deep learning architecture, b) Comparison of progression of mean F1 score across all

experiments, grouped by training mechanism, c) Comparison of progression of train-loss and test-loss across all experiments, d) Comparison of progression of mean F1 score across all experiments, grouped by train-test set splits, e) Comparison of progression of mean F1 score across all experiments, grouped by dataset type.

**Figure S2
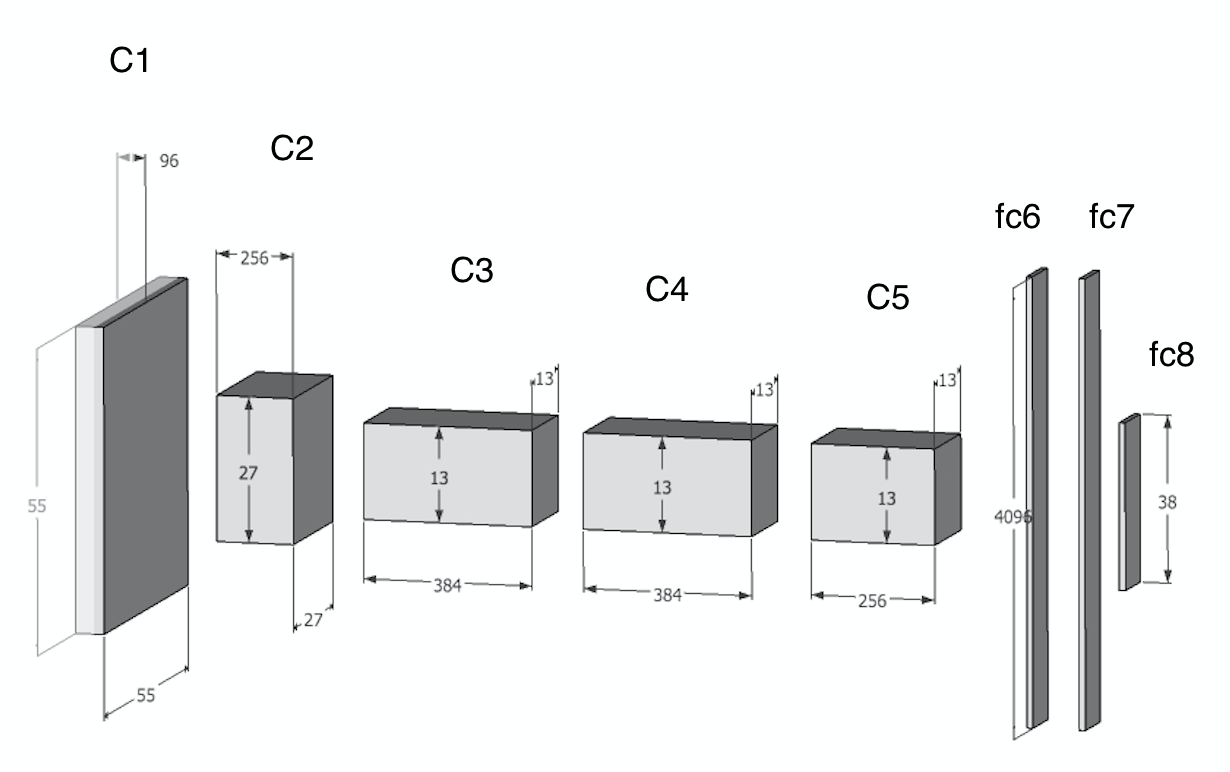
**

**Fig. S2.** Overall architecture of AlexNet as used in this study. Input layer is not shown, but consists of the raw pixels of an image across the RGB channels (i.e. dimensions 256x256x3). C{1,2,3,4} refer to a convolution layer followed by a normalization layer followed by a pooling layer. C5 refers to a convolution layer followed by a single pooling layer. fc{6,7,8} are fully connected layers. Note that the final layer fc8 is of size 38, corresponding to the 38 classes shown in Figure 1 in the main text. Starting from the input layer left of C1, the values of each layer are used as inputs to calculate - through transformation functions - the values in the next layer. The process of training the network consists of finding the optimal transformation functions that yield the best results.

**Table S1**

| *Class name (crop~disease pair)* | *Number of images in the dataset* |
| --- | --- |
| *Apple~Apple_scab* | *630* |
| *Apple~Black_rot* | *621* |
| *Apple~Cedar_apple_rust* | *275* |
| *Apple~healthy* | *1645* |
| *Blueberry~healthy* | *1502* |
| *Cherry~healthy* | *854* |
| *Cherry~Powdery_mildew* | *1052* |
| *Corn~Cercospora_leaf_spot Gray_leaf_spot* | *513* |
| *Corn~Common_rust* | *1192* |
| *Corn~healthy* | *1162* |
| *Corn~Northern_Leaf_Blight* | *985* |
| *Grape~Black_rot* | *1180* |
| *Grape~Esca_(Black_Measles)* | *1383* |
| *Grape~healthy* | *423* |
| *Grape~Leaf_blight_(Isariopsis_Leaf_Spot)* | *1076* |
| *Orange~Haunglongbing* | *5507* |
| *Peach~Bacterial_spot* | *2297* |
| *Peach~healthy* | *360* |
| *Pepper_bell~Bacterial_spot* | *997* |
| *Pepper_bell~healthy* | *1478* |
| *Potato~Early_blight* | *1000* |
| *Potato~healthy* | *152* |
| *Potato~Late_blight* | *1000* |
| *Raspberry~healthy* | *371* |
| *Soybean~healthy* | *5090* |
| *Squash~Powdery_mildew* | *1835* |
| *Strawberry~healthy* | *456* |
| *Strawberry~Leaf_scorch* | *1109* |
| *Tomato~Bacterial_spot* | *2127* |
| *Tomato~Early_blight* | *1000* |
| *Tomato~healthy* | *1591* |
| *Tomato~Late_blight* | *1909* |
| *Tomato~Leaf_Mold* | *952* |
| *Tomato~Septoria_leaf_spot* | *1771* |
| *Tomato~Spider_mites* | *1676* |
| *Tomato~Target_Spot* | *1404* |
| *Tomato~Tomato_mosaic_virus* | *373* |
| *Tomato~Yellow_Leaf_Curl_Virus* | *5357* |

**Table S1.** Number of images in data set for each of the 38 classes, as shown in figure 1 in the main text.
